# Novel RNA viruses within plant parasitic cyst nematodes

**DOI:** 10.1371/journal.pone.0193881

**Published:** 2018-03-06

**Authors:** Casey L. Ruark, Michael Gardner, Melissa G. Mitchum, Eric L. Davis, Tim L. Sit

**Affiliations:** 1 Department of Entomology and Plant Pathology, North Carolina State University, Thomas Hall, Raleigh, North Carolina, United States of America; 2 Division of Plant Sciences and Bond Life Sciences Center, University of Missouri, 371H Bond Life Sciences Center, Columbia, Missouri, United States of America; Oklahoma State University, UNITED STATES

## Abstract

The study of invertebrate–and particularly nematode–viruses is emerging with the advancement of transcriptome sequencing. Five single-stranded RNA viruses have now been confirmed within the economically important soybean cyst nematode (SCN; *Heterodera glycines*). From previous research, we know these viruses to be widespread in greenhouse and field populations of SCN. Several of the SCN viruses were also confirmed within clover (*H*. *trifolii*) and beet (*H*. *schachtii*) cyst nematodes. In the presented study, we sequenced the transcriptomes of several inbred SCN populations and identified two previously undiscovered viral-like genomes. Both of these proposed viruses are negative-sense RNA viruses and have been named SCN nyami-like virus (NLV) and SCN bunya-like virus (BLV). Finally, we analyzed publicly available transcriptome data of two potato cyst nematode (PCN) species, *Globodera pallida* and *G*. *rostochiensis*. From these data, a third potential virus was discovered and called PCN picorna-like virus (PLV). PCN PLV is a positive-sense RNA virus, and to the best of our knowledge, is the first virus described within PCN. The presence of these novel viruses was confirmed via qRT-PCR, endpoint PCR, and Sanger sequencing with the exception of PCN PLV due to quarantine restrictions on the nematode host. While much work needs to be done to understand the biological and evolutionary significance of these viruses, they offer insight into nematode ecology and the possibility of novel nematode management strategies.

## Introduction

Specific genera of plant parasitic nematodes (PPN) are non-propagative vectors of plant viruses [1], but PPN themselves were thought to be immune to viral infections. Recent advances in whole transcriptome sequencing, however, have led to the discovery of novel viruses infecting nematodes. The first nematode viruses, belonging to the family *Nodaviridae*, were identified within natural populations of *Caenorhabditis elegans* and *C*. *briggsae* [2,3]. Soon afterward, the first viruses infecting PPN were found within *Heterodera glycines* (soybean cyst nematode; SCN) belonging to families *Bunyaviridae*, *Bornaviridae*, *Rhabdoviridae*, and *Flaviviridae* [4,5]. Additionally, a novel nodavirus element has recently been described within the plant parasitic pinewood nematode, *Bursaphelenchus xylophilus* [6]. Previous research has demonstrated the widespread prevalence of the known SCN viruses within inbred and field populations as well as other *Heterodera* species including beet and clover cyst nematode [7]. In addition, we confirmed that these viruses replicate within the nematode host and are found in multiple life stages. Recently, a comprehensive paper analyzing over 220 invertebrate species provided an additional 1,445 viral genome entries into the NCBI database [8]. Included in this analysis were animal parasitic nematodes belonging to *Ascaridia*, *Ascaris*, *Romanomermis*, and other unidentified nematode genera providing 32 new viral genomes for further investigation. One over-arching commonality of nematode viruses discovered so far is that they possess single-stranded RNA genomes. These viruses were not initially discovered by genome sequencing as there is no DNA intermediate and thus, cannot be readily detected without RNA sequencing technology.

The study presented here analyzed nematode transcriptome data to identify and describe novel viral genomes via de novo assembly with the assistance of the VirFind toolset (virfind.org) [9]. Nematode species analyzed for viruses in this study include *Heterodera glycines*, *H*. *schachtii*, *H*. *avenae*, *H*. *trifolli*, *Globodera pallida*, *G*. *rostochiensis*, *G*. *tabacum*, and *Vittatidera zeaphila*. These cyst nematode species cause significant destruction to agricultural crops and are thus of economic importance [10,11]. Moreover, a limited toolset currently exists for growers to defend their crops against these pests. Identification of novel viral genomes will provide a greater understanding of nematode biology and potentially provide innovative management strategies.

## Materials and methods

### Processing of biological samples for RNAseq

Two inbred populations of *Heterodera glycines*, OP25 and OP50 [12], were cultured on roots of ‘Hutcheson’ soybean plants grown under greenhouse conditions. The SCN cysts were extracted from plant roots with water pressure and soil via water flotation and collected on nested sieves of screen sizes 20 (850 μm) and 60 (250 μm). SCN eggs were extracted by crushing cysts with a Tenbroeck homogenizer and further separated from soil debris by centrifugation through 70% sucrose and collection on a size 500 screen (25 μm). Equipment was sterilized and stored separately for each SCN population. Eggs were surface sterilized in 2% (w/v) sodium azide for 29 minutes. Eggs were hatched at 27°C using the Baermann pan method [13] and the hatched pre-parasitic second-stage juveniles (ppJ2s) were collected within 48 hours of hatch on a size 500 screen. Nematode samples were prepared for total RNA extraction by vibration homogenization for 20 seconds with 3-mm glass beads in a 1.5 ml tube on a Silamat S6 (Ivoclar Vivadent, Amherst, NY). Total RNA from approximately 10,000 pooled ppJ2s was extracted via TRIzol^®^ Reagent (Invitrogen, Carlsbad, CA) under the guidelines of the manufacturer’s protocol adapted from [14]. The nematode total RNA samples were prepared and processed for sequencing at the NC State University Genomic Sciences Laboratory (NCSU GSL; Raleigh, NC). Sample concentration was measured via the 2100 BioAnalyzer (Agilent Technologies, Santa Clara, CA). To enrich for sequence coverage of viruses, samples were treated for ribosomal depletion by preparation with NEBNext^®^ Ultra™ Directional RNA Library Prep Kit for Illumina^®^ (New England BioLabs, Ipswich, MA). OP25 and OP50 RNA were run via paired-end Illumina MiSeq 300x300bp at the NCSU GSL. Raw sequence reads are available under the NCBI Short Read Archive (SRA) accession numbers SRR6269844 and SRR6269845.

The SCN inbred population MM8 (HG Type 2.5.7) was propagated under greenhouse conditions on plant introduction 88788 [15]. Freshly hatched ppJ2 were inoculated onto 10-day old seedlings and the inoculated plants were placed in the greenhouse. Five days post-inoculation, parasitic second-stage juveniles (pJ2) nematodes were isolated from the roots by blending the roots for 30 seconds in a kitchen blender. Following this, the root homogenate was poured over a nested stack of sieves with pore sizes of 850μm, 250μm, and 25μm before purifying the nematodes from the sample using sucrose centrifugal flotation [16]. RNA was isolated from frozen nematode pellets using the PerfectPure Fibrous Tissue Kit (5Prime) and a modified version of the manufacturer’s extraction protocol [17]. RNA quality was determined using a Fragment Analyzer (Advanced Analytical) and quantified using a Qubit Fluorometer prior to library preparation. RNAseq libraries were constructed using the TruSeq mRNA Stranded Library Prep Kit (Illumina) and sequenced on the Illumina HiSeq 2500 platform in a paired-end manner (2x50). Library preparation and high-throughput sequencing services were performed at the University of Missouri DNA Core Facility. Three biological replicates of each sample were sequenced. Raw sequence reads are available under the NCBI SRA accession numbers SRR6232814-SRR623816 and SRX3341252-SRX3341254.

Transcriptome data (Roche 454) of *H*. *schachtii* J2s (SRR1125017) are publicly available on the NCBI SRA database from the University of Murdoch [18]. Transcriptome data (Illumina Genome Analyzer II) of pooled J2s and females of *H*. *avenae* are available under accession number ERR414136, and is provided by the Indian Agricultural Research Institute. *G*. *pallida* (PCN) transcriptome files (Illumina Genome Analyzer II) of males (ERR202422) and females (ERR202423) are publicly available from the Wellcome Trust Sanger Institute [19]. *G*. *rostochiensis* (PCN) female RNAseq data (Illumina HiSeq 2000) are available from the University of Dundee (ERR1173512).

### Bioinformatic analysis

RNA sequencing fastq files were uploaded onto the VirFind server [9] (virfind.org). Blastx and Blastn E-values were set at 1.0E-2 with no trimming of contigs. De novo assembled contigs were uploaded into Geneious version 9.1.7 (Biomatters, Auckland, New Zealand) [20] and contigs ≥ 3kb were further analyzed for viral signatures. The contigs were first screened for predicted open reading frames (ORFs) within Geneious software; those assemblies that had ORFs with similar organization to viruses were translated into amino acids. Translated proteins were screened for predicted function via NCBI PSI-BLAST (position-specific iterated BLAST) of non-redundant protein sequences (nr). Additional filtering to find true viral sequences was conducted with the InterProScan [21] plug-in within Geneious software. Each recovered viral genome was independently de novo assembled via VirFind.

To determine coverage of viruses within nematode datasets, genomes were reassembled in Geneious with BBMap using the VirFind de novo assembly as a reference sequence (Table 1). Phylogenetic trees were built by compiling the closest hits to the novel virus translated polymerase or polyprotein based upon NCBI PSI-BLAST. Proteins were aligned with ClustalW [22] BLOSUM cost matrix (gap open cost = 10, gap extend cost = 0.1). Trees were constructed from the protein alignment with Geneious Tree Builder using the Jukes-Cantor genetic distance model and a neighbor-joining tree method. No outgroup was selected, and the tree was resampled via bootstrap method with 1000 replicates (50% support threshold). To further characterize SCN nyami-like virus (NLV), conserved transcription initiation/termination sites were identified by aligning non-coding regions with MAAFT version 7.222 [23] (200PAM/k = 2 scoring matrix; 1.53 gap open penalty; 0.123 offset value) and selecting conserved domains with similarity to other *Nyamiviridae* [24]. Additionally, putative protease cleavage sites of PCN picorna-like virus (PLV) were predicted via the NetPicoRNA 1.0 Server (http://www.cbs.dtu.dk/services/NetPicoRNA/) [25].

**Table 1:** Number of sequence reads and mean read coverage of viral genomes from mined transcriptome data sets. Sequence coverage was determined in Geneious software version 9.1.7 with BBMap using de novo assemblies generated by VirFind [9]. Abbreviations: J2 (second-stage juvenile), SCN (soybean cyst nematode), BCN (beet cyst nematode), CCN (cereal cyst nematode), PCN (potato cyst nematode), *Gp* (*Globodera pallida*), *Gr* (*G*. *rostochiensis*), NLV (nyami-like virus), BLV (bunya-like virus), PLV (picorna-like virus). Mean describes average sequence read coverage for each nucleotide position. ND (not detected) denotes the virus was not found in the specified nematode sample.

			SCN NLV		SCN BLV		PCN PLV	
Sample	Run ref #	Reads	Reads	Mean	Reads	Mean	Reads	Mean
SCN OP25 J2s	SRR6269844	11.4 mil	22,989	557	5845	168	ND	ND
SCN OP50 J2s	SRR6269845	12.7 mil	34,351	820	5619	161	ND	ND
SCN MM8 J2s	SRR6232814-16	71.3 mil^a^	7391	50	305^b^	3	ND	ND
BCN J2s	SRR1125017	184,024	1714	3	ND	ND	ND	ND
CCN J2s + females	ERR414136	46.1 mil	ND	ND	ND	ND	ND	ND
PCN (*Gp*) males	ERR202422	33.7 mil	ND	ND	ND	ND	88,829	731
PCN (*Gp*) females	ERR202423	33.5 mil	ND	ND	ND	ND	12,138	99
PCN (*Gr*) females	ERR1173512	41.2 mil	ND	ND	ND	ND	1751	19

^a^ Read information represents three pooled biological replicates.

^b^ Sequence data spans the length of the genome; however, there is poor coverage within A-T rich regions creating several large, undermined gaps in the genome.

### Confirmation of viral presence in nematodes

Nematode eggs maintained at the University of Missouri and juveniles maintained at Cornell University and North Carolina State University were collected similarly to the process described for RNAseq. The inbreeding protocol, date of origin, and HG type of SCN populations used for this manuscript can be viewed in a previous publication from our laboratory [7]. Total RNA was extracted as described previously via an adapted TRIzol^®^ Reagent protocol (Invitrogen, Carlsbad, CA) [14]. Total RNA concentrations were analyzed via Nanodrop 1000 (Thermo Fisher Scientific, Waltham, MA). cDNA was synthesized by incubating approximately 1 μg RNA with 0.06 μg random primers (Invitrogen) for 10 minutes at 70°C followed by rapid cooling on ice. Next, 4 μl GeneAmp^®^ 10X PCR Buffer II (Applied Biosystems, Foster City, CA), 5.5 mM MgCl_2_, 0.5 μM deoxynucleotide solution mix, 32 U Murine RNase Inhibitor (New England BioLabs, Ipswich, MA), and 50 U Multiscribe^™^ Reverse Transcriptase (Applied Biosystems) were added before additional incubations of 42°C and 70°C for 15 minutes each.

All primers used for viral detection and analysis were synthesized by Eurofins Genomics (Louisville, KY) and are listed on Table 2. qRT-PCR was conducted to determine if the proposed viral genomes were detectable in nematode samples. The SCN-specific internal control genes GAPDH (Glyceraldehyde 3-phosphate dehydrogenase) [7] and HgFAR1 (*H*. *glycines* fatty acid and retinol binding protein-1) [5] were used to determine the relative quantification of viral titers. Across PPN species, 18*S* rRNA was used as an internal control as other regions tested were too variable. qRT-PCR products were amplified using 0.5 μM of each appropriate primer pair, 10 μl iTaq^™^ Universal SYBR^®^ Green Supermix (Bio-Rad, Hercules, CA), and 1 μl cDNA. Applied Biosystems QuantStudio™ 6 Flex Real-Time PCR system was used with the following settings: 95°C, 20 seconds; 95°C, 2 seconds; 60°C, 25 seconds repeated for 40 amplification cycles with a continuous melt curve of 95°C, 20 seconds; 60°C, 1 minute; and 94°C for 20 seconds.

**Table 2:** Primers used for this research study and their application. Abbreviations: NLV (nyami-like virus), BLV (bunya-like virus), PLV (picorna-like virus), SCN (soybean cyst nematode), PPN (plant parasitic nematode).

Primer	Application	Sequence [5’ to 3’]
NLV_QF	qRT-PCR	GTTGACGGCACTTGAACACC
NLV_QR	qRT-PCR	GGGATTCAACTCAAGCCGGA
NLV_ORF1_F	Endpoint PCR	GACCAAGTGCCTCGTTCTCA
NLV_ORF1_R	Endpoint PCR	CCAAAGCCATCCCGTTGTTG
NLV_ORF2_F	Endpoint PCR	CCTCTTCTTCTTCTGCCGCA
NLV_ORF2_R	Endpoint PCR	TTGTGCTTGGTGTTAACGCG
NLV_ORF3_F	Endpoint PCR	CCCAGACCGAGCCTATGAAC
NLV_ORF3_R	Endpoint PCR	GGTCACGGGAAAGGGGAATT
NLV_ORF4_F	Endpoint PCR	TTGTCTTGACACTCGCCCTC
NLV_ORF4_R	Endpoint PCR	CTCCGACATACACCGAGTCG
NLV_ORF5_F	Endpoint PCR	CAGGAGCATTCGTGATTGCG
NLV_ORF5_R	Endpoint PCR	GAGCACCGACAACTACACCA
NLV_SEQ_F	Sanger sequencing	TAGGGCCACAATTGCTCGTT
NLV_SEQ_R	Sanger sequencing	TCGTGCGGACTTCAAGACAA
BLV_QF	qRT-PCR	GCCAGCCAGCATTTACAAGG
BLV_QR	qRT-PCR	CCAGGGGACATGAGAATCACC
BLV_F	Endpoint PCR	GCTGCTTCAGATCCAACAGC
BLV_R	Endpoint PCR	GCACCAGGACCCCATTTAGT
BLV_SEQ_F	Sanger sequencing	TGGTTGTTGTGTTTCGGATCAC
BLV_SEQ_R	Sanger sequencing	GGCACCACCCCATTGAACTT
PLV_QF	qRT-PCR	ACATGCGGCCAAAACATTCC
PLV_QR	qRT-PCR	AGCGCGTCATAAGCAAATGC
SCN_HgFAR1_F	qRT-PCR	CCATTTGCCGCCTTTGGA
SCN_HgFAR1_R	qRT-PCR	GGGATCAATTCGCGGTATTCG
SCN_GAPDH_F	qRT-PCR	TCCAAGGCATAGAAAGACGACG
SCN_GAPDH_R	qRT-PCR	AACAAGTCATTGGACGGCATCA
PPN_18*S*_F	qRT-PCR	GGTAGTGACGAGAAATAACG
PPN_18*S*_R	qRT-PCR	CTGCTGGCACCAGACTTG

Cycle threshold values (Ct; amplification cycle in which fluorescence emitted exceeds background fluorescence) equal to or greater than 35 were considered non-detectable. DNase treatments yielded insignificant results between Ct values of treated and untreated samples and was not necessary for analysis. The average normalized abundance ratios (i.e. relative amount of virus in each nematode sample) were determined for each population sample. Ct values of viruses were normalized against the mean Ct values of nematode internal reference genes (18*S* or an average of GAPDH and HgFAR1) using the equation E_internal_^Ct(internal)^/E_viral_^Ct(viral)^ where E equals the efficiency of a primer pair [26]. Further modifications were made for addressing viral abundance compared with host internal control genes [7,27]. Primer efficiencies were calculated by the equation 2^(-1/slope)^ via a five-point 1:2 dilution series. The efficiencies of primer pairs are E_NLV_ = 2.01 (101%), E_BLV_ = 1.94 (94%), E_HgFAR1_ = 2.05 (105%), E_GAPDH_ = 2.07 (107%), and in *H*. *glycines* E_18*S*_ = 1.95 (95%) versus E_18*S*_ = 1.81 (81%) in *H*. *trifolii*.

To confirm positive qRT-PCR results, approximately 0.8 kb regions of viral RdRPs were amplified via endpoint PCR. OneTaq^®^ 2X Master Mix with standard buffer (New England BioLabs) and appropriate primers (Table 2) were used according to manufacturer’s protocol to amplify products from cDNA in the Bio-Rad C1000 Touch Thermal Cycler under the following conditions: 94°C, 5 minutes; 94°C, 30 seconds; 60°C, 30 seconds; 68°C, 60 seconds for 40 amplification cycles followed by a final extension of 68°C for 5 minutes. Primers pairs were designed within additional ORFs (I, II, III, and IV) of SCN NLV to demonstrate correct assembly size. Products were electrophoresed on a 2% TAE agarose gel with 1X TAE buffer. PCR products were purified for Sanger sequencing with DNA Clean & Concentrator^TM^ (Zymo Research Corp., Irvine, CA). Sanger sequencing was performed by Eurofins Genomics (Louisville, KY) via nested sequence primers listed in Table 2. Nucleotide sequences were translated and aligned with Geneious version 9.1.7 (Biomatters) using ClustalW (Blosum cost matrix, gap open cost of 10, gap extend cost of 0.1).

## Results

VirFind [9] (virfind.org) analysis of cyst nematode transcriptomes identified three novel viral genomes. The names and NCBI Genbank accession numbers for the nematode viruses identified in this report are listed in Table 3. Two of these viruses were embedded within the transcriptomes obtained from SCN populations OP25 and OP50 maintained in NC State University greenhouses [12] and SCN population MM8 maintained in University of Missouri greenhouses [15]. A nearly complete genome of nyami-like virus (SCN NLV), likely belonging to the family *Nyamiviridae*, was recovered as well as the RNA dependent RNA polymerase (RdRP) gene of a bunya-like virus (SCN BLV). SCN NLV and BLV are negative-sense RNA viruses; NLV is monopartite and BLV is most similar to known multipartite viruses. Viral genome coverage was not high in MM8 likely due to differences in RNA sample preparation and sequencing methodology. Moreover, a partial sequence related to the SCN NLV RdRP was recovered from transcriptome data of the greenhouse culture of *Heterodera schachtii* (beet cyst nematode; BCN). The viral-like RdRP sequence from BCN is approximately 1.8kb long and has 67% nucleotide identity to NLV originating from SCN. An additional viral genome was identified from *Globodera pallida* (potato cyst nematode; PCN) transcriptome data [19]. The virus is a picorna-like virus (PCN PLV) and is a positive-stranded RNA virus that typically generates a single polyprotein [28]. In addition to *G*. *pallida*, the PLV genome was also assembled from transcriptome data of the other PCN species, *G*. *rostochiensis*, at low levels. The presence of novel viruses was confirmed via qRT-PCR, endpoint PCR, and Sanger sequencing with the exception of PCN PLV. The host species with high read coverage of PCN PLV, *Globodera pallida*, could not be obtained for testing due to quarantine restrictions. To compensate for this hindrance, viral genomes were independently *de novo* assembled from multiple data sets. This same process was applied to SCN NLV and SCN BLV assemblies as well.

**Table 3:** Genbank accession numbers for partial viral genomes. De novo assemblies were generated from transcriptome data via VirFind [9]. Abbreviations: J2 (second-stage juvenile), SCN (soybean cyst nematode), BCN (beet cyst nematode), PCN (potato cyst nematode), *Gp* (*Globodera pallida*), *Gr* (*Globodera rostochiensis*), NLV (nyami-like virus), BLV (bunya-like virus), PLV (picorna-like virus), RdRP (RNA-dependent RNA polymerase).

Virus	Accession	Assembly length	Sample source	Source accession
SCN NLV	MG550265	11,736	SCN OP50 J2s	SRR6269845
	MG550266	11,733	SCN OP25 J2s	SRR6269844
	MG550267	11,728	SCN MM8 J2s	SRR6232814-16
	MG550268	1,815^b^	BCN J2s	SRR1125017
SCN BLV^a^	MG550269	9,478	SCN OP50 J2s	SRR6269845
	MG550270	9,478	SCN OP25 J2s	SRR6269844
	MG550271	9,469	SCN MM8 J2s	SRR6232814-16
PCN PLV	MG550272	9,321^c^	PCN (*Gr*) females	ERR1173512
	MG550273	9,371	PCN (*Gp*) males	ERR202422
	MG550274	9,334	PCN (*Gp*) females	ERR202423

^a^ SCN BLV resembles a multipartite virus; the recovered sequences represent the RdRP region.

^b^ Only a partial sequence of the viral-like RdRP was recovered with 67% nt identity to the virus within SCN.

^c^ The coding region was recovered with the exception of 3 nt from the 3’ end.

### SCN nyami-like virus (NLV)

**Fig 1:**
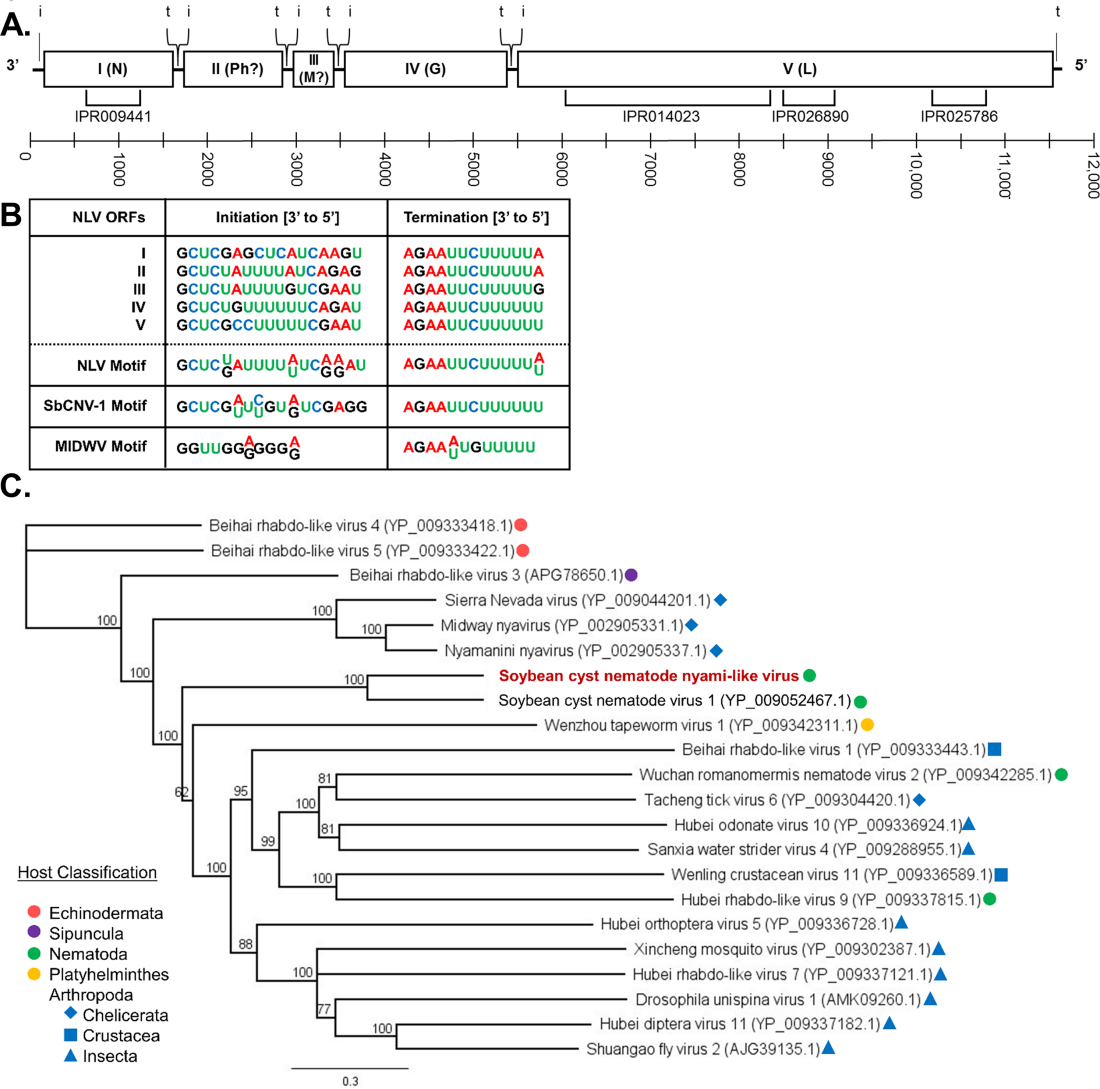
Characterization of soybean cyst nematode (SCN) nyami-like virus (NLV). (**A**) Genome organization of NLV. Putative encoded proteins are as follows: ORF I nucleocapsid (N), ORF II phosphoprotein (P), ORF III matrix protein (M), ORF IV glycoprotein (G), and ORF V RNA-dependent-RNA polymerase (RdRP) (L). Identified InterProScan regions are denoted below the genome. Locations of ORF transcription initiation (i) and termination (t) sites are shown above the genome. The scale bar represents the nucleotide length of the genome. (**B**) Initiation and termination sequences for each NLV ORF were identified by aligning non-coding regions with MAAFT version 7.222. Conserved initiation and termination motifs for NLV, soybean cyst nematode virus 1 (SbCNV-1; NC_024702.1), and Midway virus (MIDWV; FJ554525) are also provided. (**C**) Phylogenetic tree of SCN NLV RdRP in relation to RdRPs of the most closely related viruses within the NCBI database via PSI-BLAST. Proteins were aligned with ClustalW and trees constructed with Geneious Tree Builder (Jukes-Cantor genetic distance model; neighbor-joining tree method; no outgroup; 1000 replicates; 50% support threshold). Branch labels display consensus support (%).

SCN NLV is suggested to be a negative-sense RNA virus belonging to the *Mononegavirales* order and *Nyamiviridae* family. Thus far, there are three genera and five viral species within the *Nyamiviridae* family, and these viruses were isolated from invertebrate hosts [29]. The SCN NLV genome possesses five ORFs and is approximately 11.7kb in length (Fig 1A). Based upon conserved protein motifs, ORF I is 410 amino acids (AA) in length and encodes a predicted nucleoprotein—the function of which is to encapsidate the viral genome. Functions for the proteins encoded by ORFs II and III cannot definitively be determined due to lack of sequence similarity. However, when comparing SCN NLV to other *Mononegavirales*, is it likely that ORF II is a phosphoprotein (346 AA) and ORF III is a matrix protein (92 AA) [30]. Generally, phosphoproteins stabilize the RdRP to the RNA templates, and matrix proteins aid in viral particle budding. ORF IV consists of a 567 AA glycoprotein that is predicted to aid in attachment to host cells. The largest encoded protein (2060 AA) is the RdRP generated from ORF V. Notably, a single-nucleotide polymorphism (SNP) (C to U) was observed at position 5,328 of the SCN NLV sequence recovered from the SCN MM8 transcriptome data. The SNP alters the codon sequence to UUG, a start codon, and could potentially extend the RdRP protein by 4 amino acids (MCKS). From these results, conclusions cannot be drawn about the frequency of this mutation or if there is truly any effect on protein size or virus function; however, this discrepancy may be worth exploring in the future. Additionally, putative transcription initiation and termination sequences for each ORF have been identified within non-coding regions of SCN NLV (Fig 1B). The predicted initiation sequences of SCN NLV are similar for each ORF while the termination sequences are nearly identical. Overall, the conserved initiation motif is highly similar to that found in SCN virus 1 (SbCNV-1); midway virus (MIDWV), a member of the same family, is also similar but possesses a truncated sequence with six fewer nucleotides. The termination motifs for SCN NLV, SbCNV-1, and MIDWV were nearly identical to one another. This relatedness provides additional support for the placement of SCN NLV within the *Nyamiviridae* family.

SCN NLV, is most closely related to SbCNV-1 (Fig 1C) but does not belong to the *Socyvirus* genus according to a proposal describing the *Nyamiviridae* family [29]. Criteria for inclusion of SCN NLV within the *Socyvirus* genus requires the full-length genome sequence be <30% different from SbCNV-1. The genome sequences of SbCNV-1 and SCN NLV differ by approximately 50% suggesting that SCN NLV either requires a new genus for classification or the species definition will need to be expanded. The protein AA identity of NLV compared to SbCNV-1 ranges from 55.1% (RdRP; ORF V) to 23.5% (putative phosphoprotein; ORF II). The virus with the second-most similarity to SCN NLV (after SbCNV-1) is the tick-transmitted nyamanini nyavirus (35% genome identity), which is also the type species for the *Nyamiviridae* family. SCN NLV is similar to a number of invertebrate viruses, many of which come from a single, large-scale study [8]. Many of these related viruses were isolated from other nematode species (non-plant pathogens), a tapeworm species, and a range of arthropod hosts including those belonging to *Chelicerata*, *Crustacea*, and *Insecta*.

### SCN bunya-like virus (BLV)

**Fig 2:**
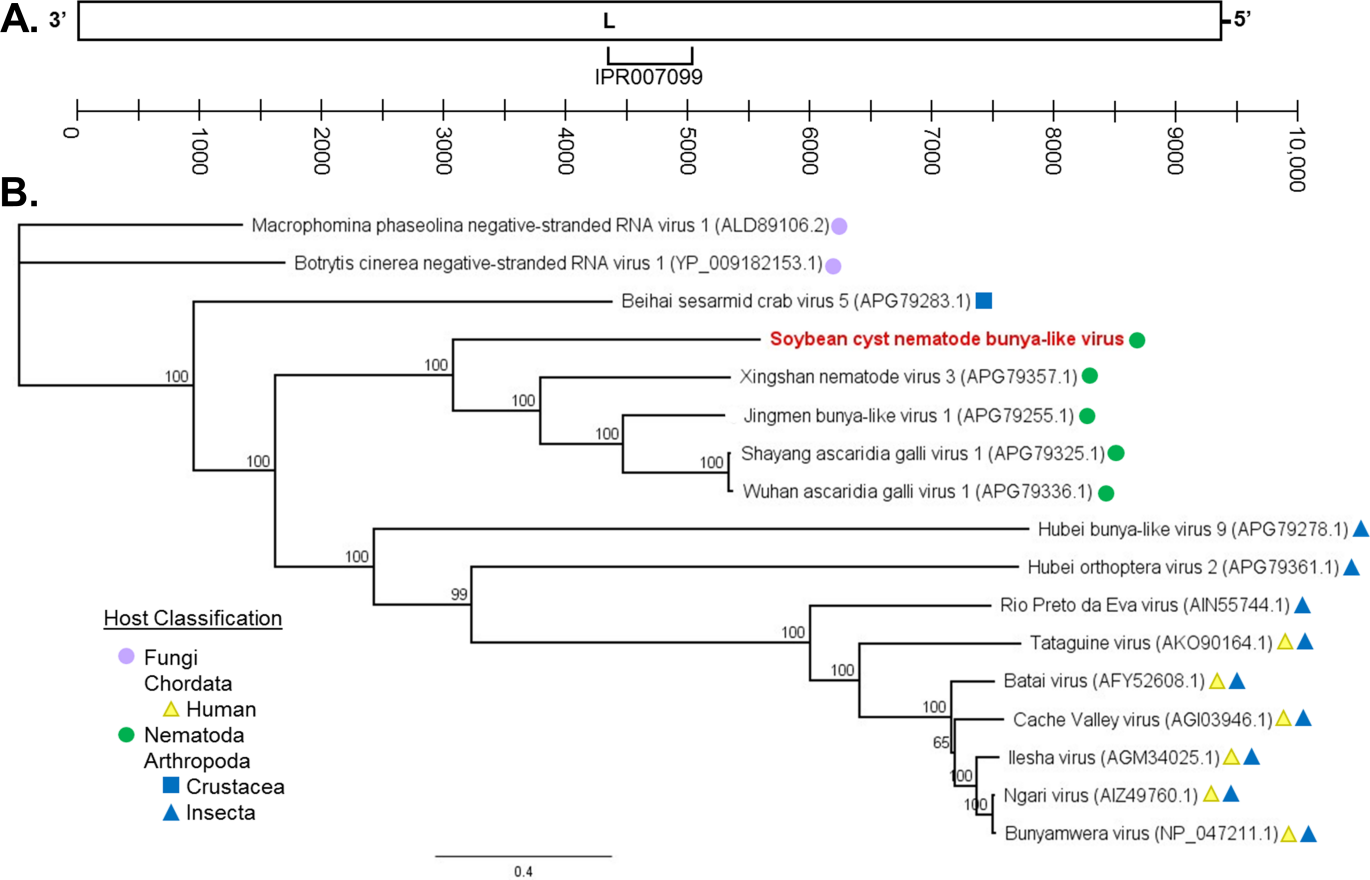
Characterization of soybean cyst nematode (SCN) bunya-like virus (BLV). (**A**) RNA-dependent RNA polymerase (RdRP; L) of SCN BLV. An identified InterProScan region is shown below the ORF. The scale denotes nucleotide length. (**B**) Phylogenetic tree of SCN BLV RdRP in relation to polymerases of closely related viruses via NCBI PSI-BLAST. Proteins were aligned with ClustalW and trees constructed with Geneious Tree Builder (Jukes-Cantor genetic distance model; neighbor-joining tree method; no outgroup; 1000 replicates; 50% support threshold). Branch labels display consensus support (%).

SCN BLV is proposed to be a multipartite, negative-sense RNA virus belonging to the order *Bunyavirales*. The RdRP portion of the genome was recovered and used for bioinformatic analysis of transcriptome data (Fig 2A). Incomplete non-coding regions were also recovered on either side of the RdRP ORF. The ORF of the RdRP is approximately 9.45 kb long and produces a protein 3150 AA in length. This virus was identified within SCN transcriptome data for OP25, OP50, and MM8. The RdRP protein of SCN BLV appears truncated from OP25 and MM8 samples (3140 versus 3149 AA). However, this is probably a result of poor read coverage of the terminal end, and the ORF likely extends to the same point as the assembly originating from OP50. Much like SCN NLV, viruses most closely related to SCN BLV infect other nematode species and arthropods–specifically crustaceans and insects (Fig 2B) [8]. The polymerase of SCN BLV groups with four other viruses originating from nematode hosts. The most closely related viral RdRP protein, Xingshan nematode virus 3 (APG79357), is 27.44% identical and originates from a mixture of *Spirurian* nematodes. The classifications of these nematode viruses has not yet been determined, and so it is difficult to predict where SCN BLV belongs beyond the Order taxon. Although SCN BLV and the most closely related nematode viruses vary greatly from one another, conserved protein motifs were identified to correctly characterize the genome. An alignment of the RdRP with that of other related viruses shows conserved motifs as demonstrated by Donaire *et al*. [31] for Bunya-like viruses (S1 Fig).

### PCN picorna-like virus (PLV)

**Fig 3:**
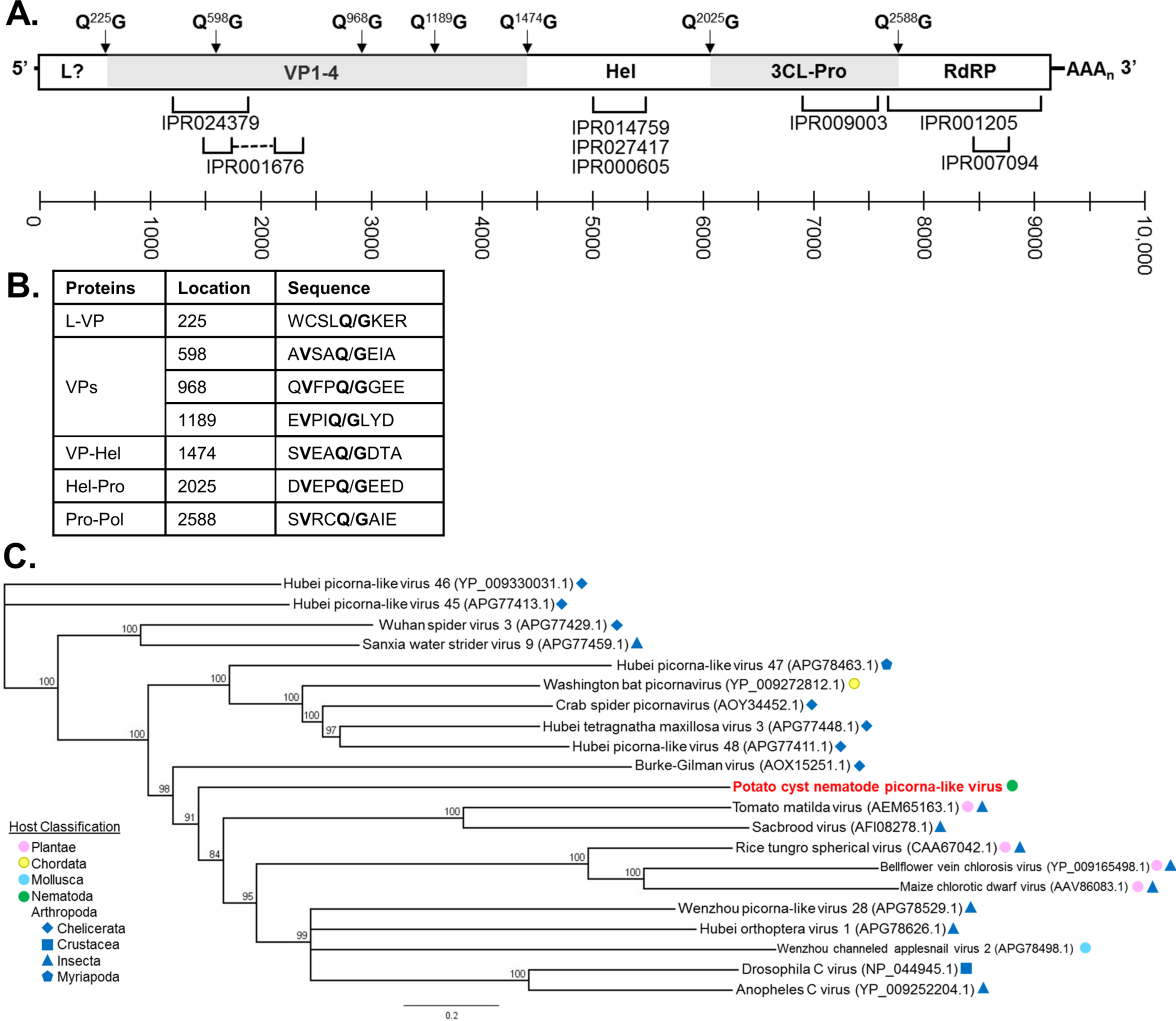
Characterization of potato cyst nematode (PCN) picorna-like virus (PLV). (**A**) Genome organization of PCN PLV. Putative proteins cleaved from the polyprotein include leader (L), four viral particle (VP1-4) proteins, helicase (hel), 3C-like protease (3CL-Pro), and RNA-dependent RNA polymerase (RdRP). Putative protease cleavage sites are shown above the genome (-1 and +1 positions); InterProScan regions are below the genome. The scale represents nucleotide length of the genome. (**B**) Putative protease cleavage sites of PCN PLV as predicted by the NetPicoRNA 1.0 Server (http://www.cbs.dtu.dk/services/NetPicoRNA/). Conserved amino acids are in bold type. (**C**) Phylogenetic tree of PCN PLV polyprotein in relation to closely related viral polyproteins identified via NCBI PSI-BLAST. Polyproteins were aligned with ClustalW and trees constructed with Geneious Tree Builder (Jukes-Cantor genetic distance model; neighbor-joining tree method; no outgroup; 1000 replicates; 50% support threshold). Branch labels display consensus support (%).

A novel potential viral genome was also identified from PCN transcriptome data of the species *Globodera pallida* and *G*. *rostochiensis*. The virus likely belongs within *Picornavirales*, has a positive-sense RNA genome of approximately 9.4 kb, and produces a singular predicted polyprotein (3090 AA) (Fig 3A). There is likely a large portion of 5’ untranslated region (UTR) unrecovered from the transcriptome data as picorna-like viruses typically have 5’ UTRs upwards of 0.5 kb that contain an internal ribosome entry site (IRES) [28]. Putative cleavage sites of the polyprotein were computationally predicted via the NetPicoRNA 1.0 Server [25] and have not been experimentally verified (Fig 3B). Furthermore, these sites were compared to those mentioned in other reports characterizing *Picornavirales* cleavage sites [32–34]. The predicted regions have a conserved Q/G (glutamine/glycine) cleavage site as well as a valine (V) in the -4 position for each site except between the leader (L) and viral particle (VP) proteins. Without further experimentation, it is not clear whether this virus truly has an L protein that can sometimes be found within *Picornavirales* species [35].

PCN PLV is distantly related to other NCBI database entries (Fig 3C). The order of encoded cleaved proteins is similar to that of *Iflaviridae*; however, there is not enough resolution in the phylogenetic tree to determine the genus at this time. The most closely related virus, Burke-Gilman virus (AOX15251) [36], has a polyprotein that is 13.61% identical followed by Hubei picorna-like virus 46 (YP_009330031) [7] at 13.49% identity. Interestingly, both of these viruses were isolated from spiders. PCN PLV does not clearly group with other viruses in the database and may ultimately require its own genera for proper classification. Conserved motifs of picorna-like viruses were identified for the protease, helicase, and RdRP (S2 Fig) [37,38]. The helicase alignment shows the most dissimilarity; however, much of this occurrence can be attributed to the plant viruses: rice tungro spherical virus (RTSV), bellflower vein chlorosis virus (BVCV), and maize chlorotic dwarf virus (MCDV).

### Confirmation of viral presence in nematodes

To demonstrate whether these newly assembled viral genomes truly exist in nature, qRT-PCR analysis was conducted on eggs of 17 SCN research populations maintained at the University of Missouri. SCN NLV was detectable in 12 of these samples (71%) at levels ranging from 7.26-fold lower than the SCN internal controls to 3.12-fold higher (Table 4). Moreover, SCN BLV was detectable in 15 populations (88%) with consistently higher titers than SCN NLV. SCN BLV relative titers were primarily present at a level higher than the internal controls and spanned 1.18-fold lower to 5.44-fold higher. The prevalence of these viruses across SCN populations of different type and location demonstrates that the RNAseq generated assemblies are not artifacts and suggests a potential importance to nematode biology.

**Table 4:** qRT-PCR Ct values and relative titers of soybean cyst nematode (SCN) viruses within SCN research populations. Mean qRT-PCR cycle threshold (Ct) values are shown for SCN nyami-like virus (NLV), SCN bunya-like virus (BLV), and internal SCN control (HgFAR1 and GAPDH). Relative viral titers were calculated and log_2_ adjusted by comparison against internal controls using a modified Pfaffl method [7,26,27]. Negative log_2_ relative titers denote a value below the internal control; whereas, positive values represent titers higher than the internal controls. ND (not detected) demonstrates that virus was not found in the sample. Experiments were conducted in technical triplicates and standard deviations are shown.

	SCN NLV		SCN BLV		HgFAR1	GAPDH
SCN Population	Mean Ct	Rel titer (log_2_)	Mean Ct	Rel titer (log_2_)	Mean Ct	Mean Ct
**MM3**	23.66 ± 0.075	**0.54**	21.09 ± 0.089	**4.21**	21.76 ± 0.047	23.96 ± 0.023
**MM4**	27.01 ± 0.181	**2.27**	27.57 ± 0.114	**3.12**	27.00 ± 0.009	28.77 ± 0.093
**MM7**	29.26 ± 0.114	**-0.94**	28.75 ± 0.235	**1.04**	27.00 ± 0.053	27.57 ± 0.078
**MM8**	18.75 ± 0.042	**1.86**	20.62 ± 0.140	**1.03**	18.81 ± 0.035	20.4 ± 0.199
**MM16**	ND	**ND**	19.88 ± 0.045	**0.97**	18.37 ± 0.056	19.57 ± 0.061
**MM18**	ND	**ND**	22.98 ± 0.086	**1.48**	21.43 ± 0.270	22.97 ± 0.189
**MM19**	30.11 ± 0.066	**-7.26**	21.86 ± 0.056	**2.17**	20.84 ± 0.061	22.66 ± 0.036
**MM21**	ND	**ND**	25.73 ± 0.027	**1.80**	24.63 ± 0.048	25.68 ± 0.029
**PA3**	23.97 ± 0.024	**0.97**	22.66 ± 0.035	**3.45**	22.78 ± 0.056	24.61 ± 0.067
**TN2**	22.97 ± 0.138	**0.74**	ND	**ND**	21.89 ± 0.067	23.36 ± 0.050
**TN6**	ND	**ND**	ND	**ND**	25.91 ± 0.064	28.4 ± 0.003
**TN12**	30.54 ± 0.188	**2.62**	31.92 ± 0.196	**2.86**	30.34 ± 0.278	32.56 ± 0.478
**TN13**	20.74 ± 0.037	**3.12**	26.35 ± 0.095	**-1.18**	21.92 ± 0.080	23.52 ± 0.058
**TN14**	26.43 ± 0.095	**2.10**	28.21 ± 0.168	**1.74**	26.58 ± 0.046	27.97 ± 0.164
**TN19**	28.41 ± 0.152	**1.65**	27.01 ± 0.018	**4.44**	26.62 ± 0.061	29.68 ± 0.064
**TN20**	ND	**ND**	27.36 ± 0.147	**5.44**	28.89 ± 0.049	30.82 ± 0.341
**TN21**	19.25 ± 0.074	**1.89**	19.06 ± 0.068	**3.05**	18.80 ± 0.031	21.01 ± 0.035

In addition to SCN populations, other cyst nematode species were tested for viruses via qRT-PCR. From the limited population size tested, SCN NLV and SCN BLV were detectable in clover cyst nematode (*H*. *trifolli*) at levels comparable to the SCN population MM8 (Table 5). PCN PLV was not detectable with qRT-PCR within an available *G*. *rostochiensis* RNA sample; *G*. *rostochiensis* and *G*. *pallida* isolates could not be acquired as a result of quarantine restrictions. It is important to note that relative titers between Table 4 and Table 5 should not be directly compared as the internal controls that were utilized are different. Despite a large difference in 18*S* Ct values when compared to viruses, it was necessary to use 18*S* as a control across cyst nematode species as GAPDH and HgFAR1 primers did not work well outside of SCN.

**Table 5:** qRT-PCR Ct values and relative titers of cyst nematode viruses within plant parasitic nematode (PPN). Mean qRT-PCR cycle threshold (Ct) values are shown for soybean cyst nematode (SCN) nyami-like virus (NLV), SCN bunya-like virus (BLV), potato cyst nematode (PCN) picorna-like virus (PLV), and internal PPN control (18*S*). Relative viral titers were calculated and log_2_ adjusted by comparison against internal control using a modified Pfaffl method [7,26,27]. Negative log_2_ relative titers denote a value below the internal control; whereas, positive values represent titers higher than the internal controls. ND (not detected) demonstrates that virus was not found in the sample. Experiments were conducted in technical triplicates and standard deviations are shown. Abbreviations: J2 (second-stage juvenile), CU (Cornell University), MU (University of Missouri), NCSU (North Carolina State University).

			SCN NLV		SCN BLV		PCN PLV	PPN 18S
PPN species	Life stage	Location	Mean Ct	Rel titer (log_2_)	Mean Ct	Rel titer (log_2_)	Mean Ct	Mean Ct
*Globodera rostochiensis*	J2	CU	ND	**ND**	ND	**ND**	ND	14.03 ± 0.199
*Globodera tobacum*	egg	MU	ND	**ND**	ND	**ND**	ND	9.37 ± 0.085
*Heterodera glycines* (MM8)	egg	MU	21.19 ± 0.191	**-15.10**	21.20 ± 0.033	**-11.78**	ND	8.80 ± 0.311
*Heterodera schachtii*	J2	NCSU	ND	**ND**	ND	**ND**	ND	16.04 ± 0.058
*Heterodera trifolii*	egg	MU	25.51 ± 0.096	**-15.59**	25.55 ± 0.034	**-14.32**	ND	11.80 ± 0.055
*Vittatidera zeaphila*	egg	MU	ND	**ND**	ND	**ND**	ND	12.27 ± 0.075

PCR primers were designed within the five ORFs of SCN NLV, and the resulting products (from SCN population MM8) are the anticipated size based upon the predicted RNAseq assembly (S3A Fig): ORF I (405 bp), ORF II (327 bp), ORF III (181 bp), ORF IV (448 bp), and ORF V (838 bp). The PCR products of ORF V (viral RdRP) were purified and Sanger sequenced; likewise, sequencing was conducted on SCN BLV RdRP products of approximately the same size. Nucleotides were translated into amino acids and aligned for SCN NLV (S3B Fig) and SCN BLV (S3C Fig). SCN NLV was Sanger sequenced from ten different SCN populations as well as clover cyst nematode. There are six sites where single nucleotide polymorphisms (SNPs) are present (99.2% identical) resulting in a single amino acid variation from one SCN sample (TN21). SCN BLV was sequenced from eight different SCN populations and clover cyst nematode. SCN BLV has 48 SNPs within the sequenced region of the RdRP (94.2% identical) resulting in four sites of amino acid variation. qRT-PCR detection and Sanger sequencing of these viral RdRPs confirms that these novel SCN viruses exist in nature. Furthermore, these viruses can be detected in the majority of cultured SCN research populations tested as well as in a second species, clover cyst nematode.

## Discussion

This report describes three novel RNA viruses within cyst nematodes. Within SCN, two new negative-stranded RNA viruses have been identified. In addition, a proposed positive-stranded RNA virus was recovered from existing PCN data. To the best of our knowledge, this is the first viral genome identified from PCN. The use of the VirFind [9] application to mine cyst nematode transcriptome data that was generated in-house or publicly available was key to the identification of these three new nematode viruses. Viral infection of cyst nematodes demonstrates the ecological complexity within an agricultural system. This host-pathogen interaction includes a nematode parasitizing a plant while being infected with a combination of viruses that have currently unknown functions. To date, the viruses reported within nematodes [2–8] are all single-stranded RNA viruses but can contain quite different genome organizations.

SCN NLV was assembled from three sets of SCN J2 transcriptome data (OP25, OP50, and MM8) and partially assembled from BCN. If improved read coverage was available for BCN, it is probable that the viral-like segment identified may belong to an unidentified, but closely related, viral species due to sequence dissimilarity. SCN NLV is a potential sixth member of the family *Nyamiviridae* and is most closely related to viruses which infect nematodes and arthropods (Fig 1C). Additionally, the polymerase of a bunya-like virus (SCN BLV) was extracted from SCN J2 transcriptome data for OP25, OP50, and MM8. Recovery of the full genomes of multipartite viruses can be conducted via repeats on terminal, non-coding ends. However, the terminal sequences of the SCN BLV RdRP segment could not be assembled. Placement of SCN BLV within a genus is difficult as related viruses are primarily unclassified. However, four viruses exist which show some similarity to SCN BLV and originate from nematode hosts (Fig 2B). Both SCN NLV and SCN BLV were detected in SCN research populations and a clover cyst nematode sample via qRT-PCR, endpoint PCR, and Sanger sequencing (Tables 4 and 5, S3 Fig). SCN NLV and SCN BLV appear to be prevalent in research SCN populations, infecting 71% and 88% of samples tested, respectively. It is possible with more extensive testing of cyst nematode species that these viruses, or variants of these viruses, will be detected. Finally, a new picorna-like virus was recovered from three PCN transcriptome data sets: *G*. *pallida* (males and females) and *G*. *rostochiensis* (females). This is a positive-sense virus that encodes a single polyprotein which undergoes proteolytic cleavage (Fig 3A and 3B). PCN PLV aligns most closely with arthropod viruses, and it will likely belong to an undescribed genus as it is distantly related to other NCBI Genbank entries (Fig 3C). Unfortunately, we were unable to sufficiently test PCN species for virus via qRT-PCR and Sanger sequencing due to quarantine measures against these nematodes.

It is unclear exactly how and when these nematodes originally became infected with viruses; preliminary testing of soybean plants did not reveal the presence of these viruses. Viral infection is prevalent within tested SCN populations (Table 4), and it is probable that samples can appear virus-free but simply possess titers below the detectable limits of qRT-PCR. Likewise, viruses were detected in both egg and juvenile life stages suggesting vertical transmission is a possible route of infection. This is a similar result to what was observed in previously discovered SCN viruses [7] as these viruses are present and actively replicate within multiple life stages (including egg and J2) of the nematode. These groups of RNA viruses do not have a DNA intermediary stage, and therefore, should not be capable of integrating into the host genome. Instead, it is plausible that viruses are maternally transmitted through eggs and could be transmitted paternally as well. As this is an emerging field of research, vertical transmission has not been well studied for nematode viruses; however, it has been evidenced for viral endosymbionts of insects [39]. These SCN viruses appear to have a narrow host range, and thus, may have evolved alongside *Heterodera spp*., specifically SCN (*H*. *glycines*) and *H*. *trifolii*. Within the *Heterodera spp*., *H*. *trifolii* and *H*. *mediterranea* (not tested) are the most closely related to SCN [40].

This study contributes further knowledge to the new and evolving field of nematode virology. With additional mining of transcriptome data using appropriate informatics tools such as VirFind [9], it is likely that more viruses will be discovered within more nematode species. Elucidating the role of nematode viruses may provide insight into nematode biology and result in additional forms of pest control. Future work will focus on the impact viruses have on the nematode host, localization and transmission of viruses in nematodes, and explore the potential applications of viral infection of nematodes.

## Supporting information

S1 Fig Conserved protein motifs of soybean cyst nematode (SCN) bunya-like virus (BLV). Most closely related RNA-dependent RNA polymerase (RdRP) sequences were identified with NCBI PSI-BLAST and aligned with ClustalW protein alignment. Conserved protein motifs identified by [31] are shown above the alignments.
(TIF)

S2 Fig Conserved protein motifs of potato cyst nematode (PCN) picorna-like virus (PLV). Most closely related viruses were identified via NCBI PSI-BLAST. Proteins were aligned with ClustalW. Conserved picorna-like virus motifs were described in [37,38] and are shown above the sequence alignments. Motifs are identified within (**A**) protease, (**B**) helicase, and (**C**) RNA-dependent RNA polymerase (RdRP).
(TIF)

S3 Fig Amplification of viral products and alignments of translated soybean cyst nematode (SCN) nyami-like virus (NLV) and SCN bunya-like virus (BLV) RNA-dependent RNA polymerases (RdRPs) via Sanger sequencing. (**A**) PCR products of SCN NLV ORFs isolated from SCN population MM8. Fragments sizes are 405 bp (ORF I), 327 bp (ORF II), 181 bp (ORF III), 448 bp (ORF IV), and 838 bp (ORF V).Viral products were amplified from total RNA of SCN MM8 and electrophoresed on a 2% gel with 100 bp molecular ladder (New England BioLabs). (**B**) Amino acid alignment of SCN NLV RdRP fragments. Nucleotides were translated from Sanger sequencing results and aligned via Geneious software (Biomatters) using ClustalW (Blosum62, threshold of 4 is represented). The sequence from SCN population OP50 was obtained via Next Gen sequencing and acts as a reference for comparison. (**C**) Amino acid alignment of SCN BLV RdRP fragments. Nucleotides were translated from Sanger sequencing results and aligned via Geneious software using ClustalW (Blosum62, threshold of 4 is represented). The sequence from SCN OP50 was obtained via Next Gen sequencing and acts as a reference for comparison.
(TIF)
