# Supplementary figures and images for: Novel RNA viruses within plant parasitic cyst nematodes
Source: PLoS One. 2018 Mar 6;13(3):e0193881. doi: 10.1371/journal.pone.0193881 (PMC5839581; doi:10.1371/journal.pone.0193881)

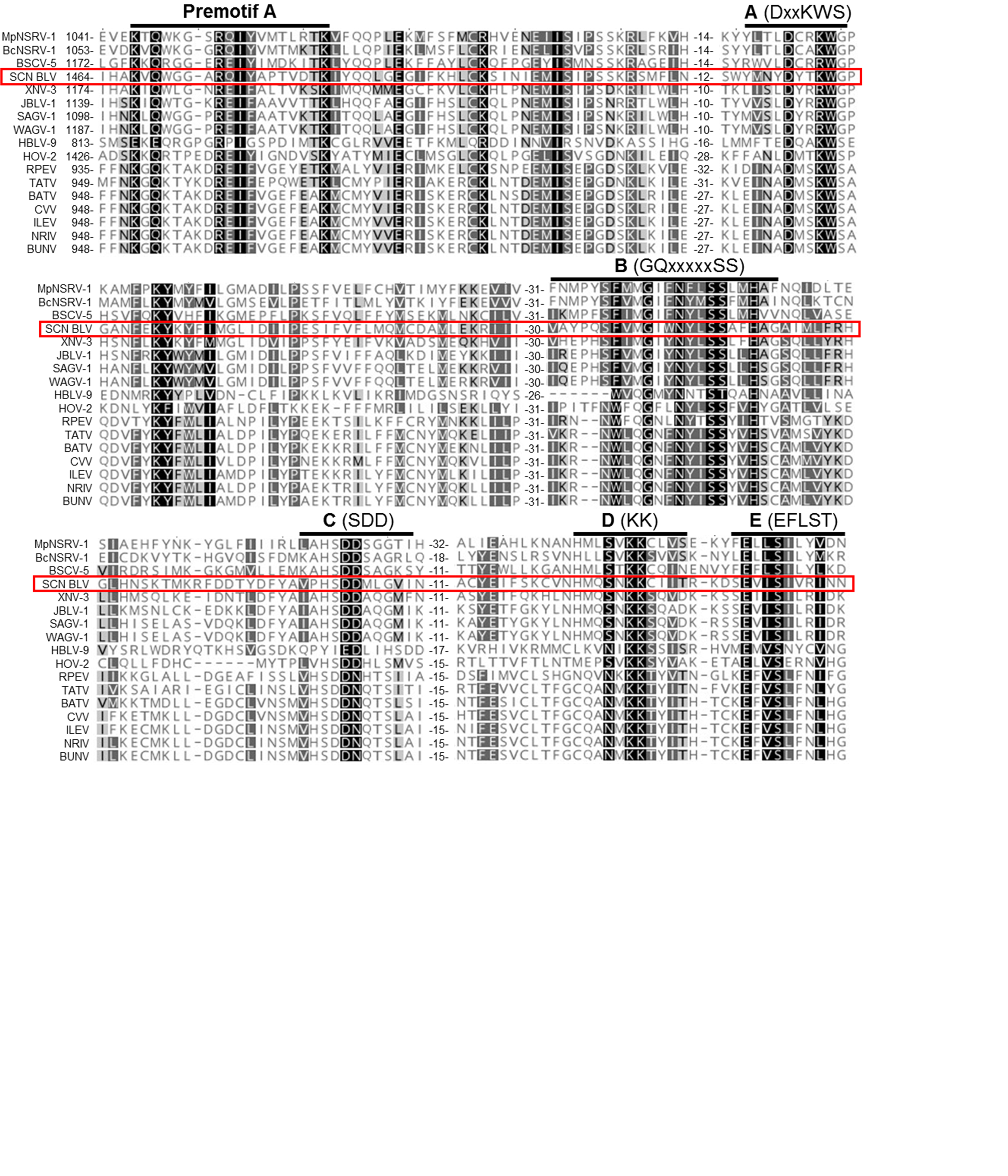

Supplement: S1 Fig — Most closely related RNA-dependent RNA polymerase (RdRP) sequences were identified with NCBI PSI-BLAST and aligned with ClustalW protein alignment. Conserved protein motifs identified by [31] are shown above the alignments. (TIF) [file pone.0193881.s001.TIF]

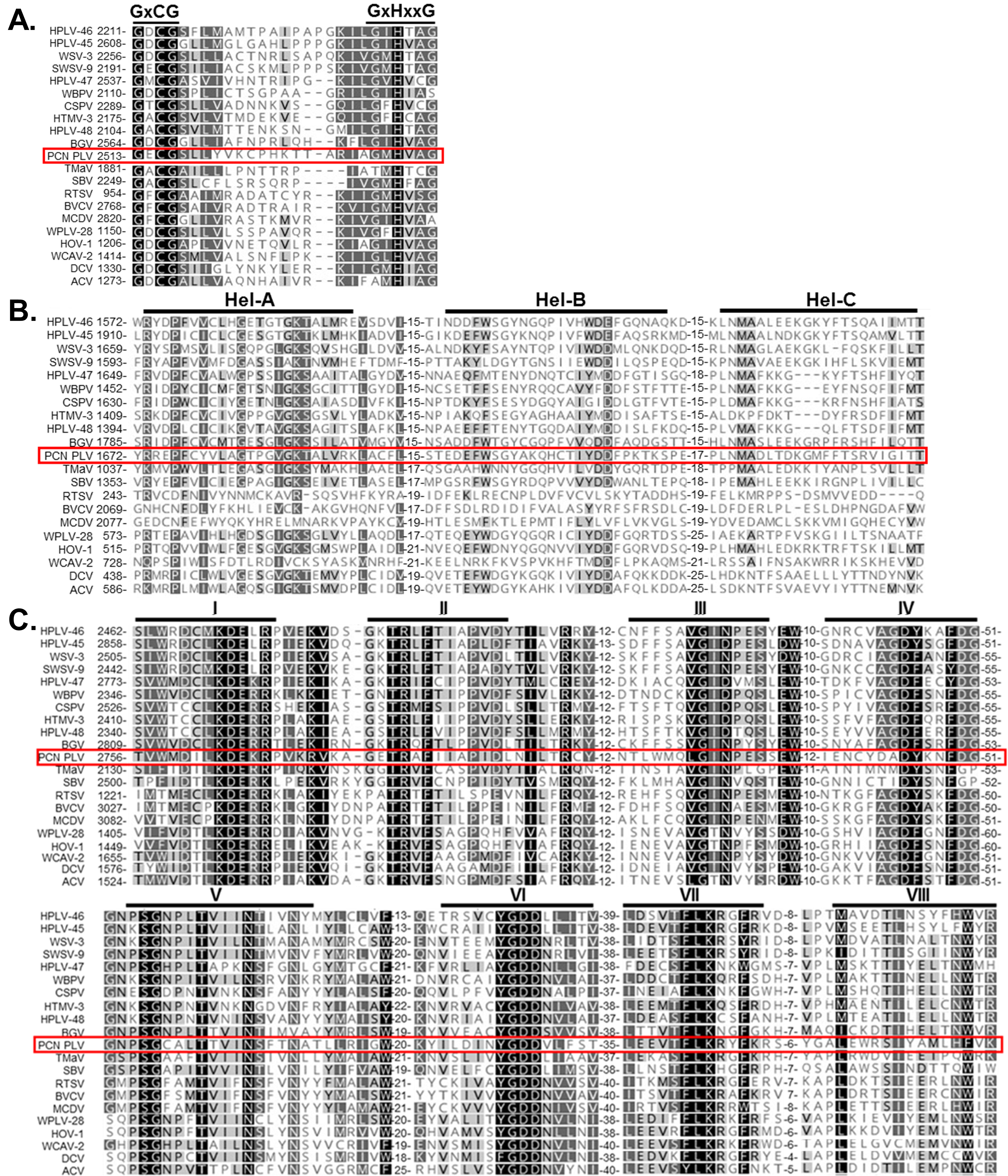

Supplement: S2 Fig — Most closely related viruses were identified via NCBI PSI-BLAST. Proteins were aligned with ClustalW. Conserved picorna-like virus motifs were described in [37,38] and are shown above the sequence alignments. Motifs are identified within (A) protease, (B) helicase, and (C) RNA-dependent RNA polymerase (RdRP). (TIF) [file pone.0193881.s002.TIF]

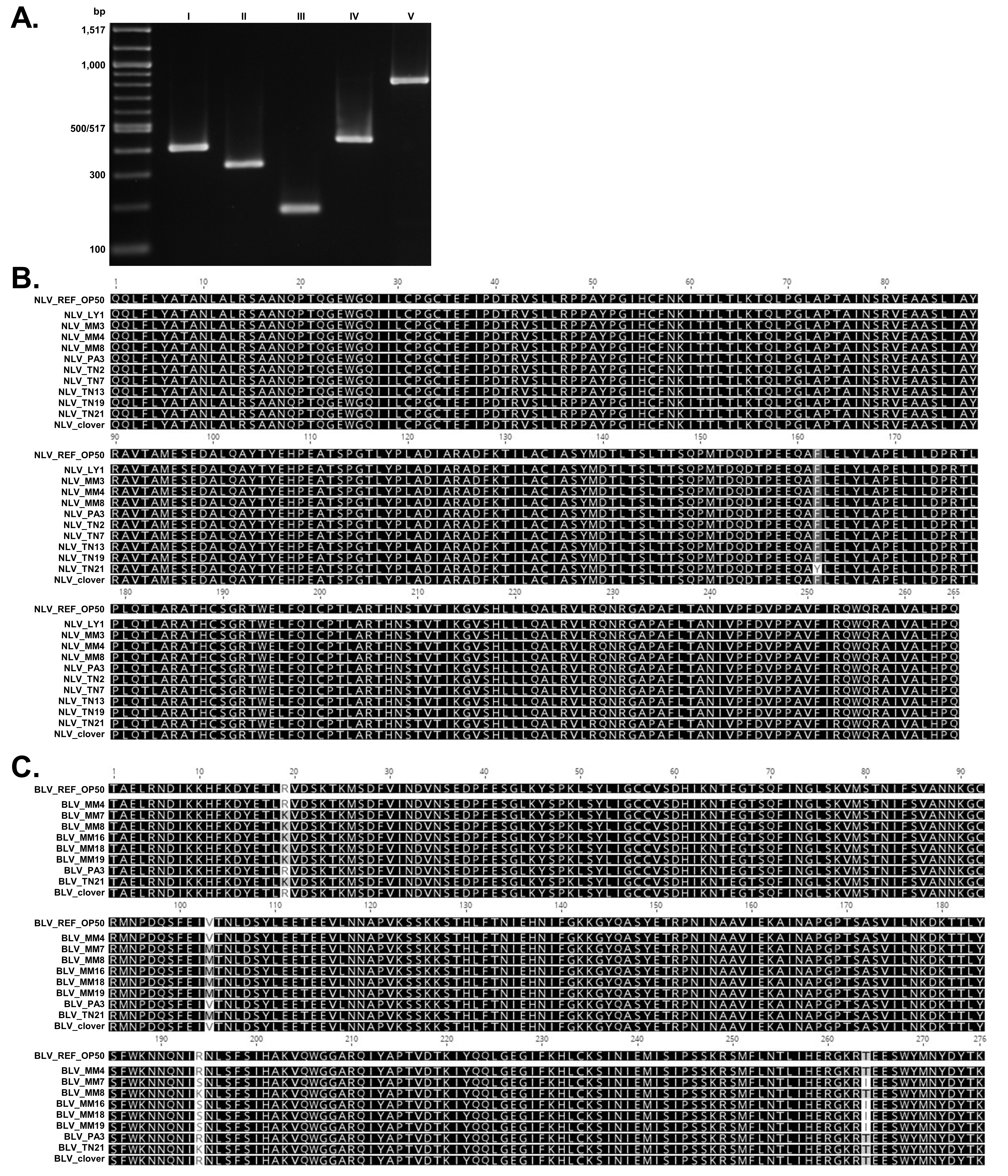

Supplement: S3 Fig — (A) PCR products of SCN NLV ORFs isolated from SCN population MM8. Fragments sizes are 405 bp (ORF I), 327 bp (ORF II), 181 bp (ORF III), 448 bp (ORF IV), and 838 bp (ORF V).Viral products were amplified from total RNA of SCN MM8 and electrophoresed on a 2% gel with 100 bp molecular ladder (New England BioLabs). (B) Amino acid alignment of SCN NLV RdRP fragments. Nucleotides were translated from Sanger sequencing results and aligned via Geneious software (Biomatters) using ClustalW (Blosum62, threshold of 4 is represented). The sequence from SCN population OP50 was obtained via Next Gen sequencing and acts as a reference for comparison. (C) Amino acid alignment of SCN BLV RdRP fragments. Nucleotides were translated from Sanger sequencing results and aligned via Geneious software using ClustalW (Blosum62, threshold of 4 is represented). The sequence from SCN OP50 was obtained via Next Gen sequencing and acts as a reference for comparison. (TIF) [file pone.0193881.s003.tif]
